# Consumption of Medicaments in the Slovak Republic

**Published:** 2017-07

**Authors:** Pavol BENO, Martin SAMOHYL

**Affiliations:** 1. Dept. of Laboratory Medicine, Faculty of Health Sciences and Social Work, Trnava University, Trnava, Slovak Republic; 2. Institute of Hygiene, Faculty of Medicine, Comenius University, Bratislava, Slovak Republic

## Dear Editor-in-Chief

High consumption of medicaments places a heavy burden on public health systems. Several major national surveys have documented important increases in the misuse and abuse of several prescription drugs (1). Analysing consumption and prescription of drugs have been conducted in several studies (2, 3).

This study aimed to analyze the consumption of individual classes of medicaments in the Slovak Republic between 2013 and 2015.

We conducted a descriptive analysis. Medicaments consumption data were obtained from the National Health Information Centre of the Slovak Republic (4–6). Consumption of medicaments was transformed to relative percentage annual difference and averaged, thus we get so-called mean percentage difference (MPD) characteristic of annual growth consumption of medicaments, formally;

$$\mathrm{MPD}=\frac{1}{n}\sum \frac{x_{i}-x_{i-1}}{x_{i=1}}*100$$

Where ***x*** represents input vector of medicaments consumption with period ***n*** in years ***i***, in each analyzed of the periods.

Each person residing in Slovakia must be by law insured by one of the health insurance companies. At present, there are three health insurance companies operating in Slovakia. In addition, medication dispensed on prescription are either fully or partially covered by health insurance to which every employed person contributes 4% of their gross salary.

In Dec 2011, a new law in Slovakia came into force that aimed to introduce mandatory generic prescribing. Doctors no longer prescribe the name of the drug but rather the name of the medication’s active substance. The patient then has the opportunity to choose a generic medicament with the lowest possible supplementary charge based on a pharmacist’s recommendation. Significant change is a financial benefit to patients. The physician is required to provide the name of the active substance when writing a prescription as well as inform the patient of the prices on all substitute generic medicines and supplementary charges for these drugs.

From a financial point of view, consumption of medications has risen since 2008. The largest share in the number of packages dispensed consists of medicines dispensed on prescriptions that are covered fully or partially by health insurance (51.3%). While the amount of packaging of prescription drugs in this period has decreased, their reimbursement from health insurance companies has been rising since 2012. In 2015, 83.6 million packages of medicines were dispensed on prescription in which the health insurance companies paid out €875.4 million (6). In 2015, the most frequently prescribed medicaments, according to the number of packages, were medications to treat the cardiovascular system (38.4 mi), the nervous system (14.1 mi) and the digestive tract and metabolism (8.9 mi). During this period, the highest increase in the number of dispensed MPD packages was observed in antineoplastic agents and immunomodulators (MPD 4.7%), meanwhile the largest decrease was found in medications to treat the cardiovascular system (MPD −2.0%) (Table 1).

**Table 1:** Medicaments consumption according to selected groups of medicaments and MPD, 2013–2015

**Medication class**	**2013 (No in Mill)** **(%)**	**2014 (No in Mill)** **(%)**	**2015 (No in Mill)** **(%)**	MPD^*^ (%)
Digestive tract and metabolism	8.68 (10.4)	8.72 (10.6)	8.96 (10.9)	2.2
Blood and blood-forming organs	4.73 (5.7)	4.84 (5.9)	4.80 (5.8)	0.8
Cardiovascular system	29.58 (35.7)	28.79 (35.0)	28.38 (34.5)	−2.0
Dermatology	3.16 (3.8)	3.18 (3.9)	3.12 (3.8)	−0.7
Urogenital system and sex hormones	1.69 (2.0)	1.66 (2.0)	1.67 (2.0)	−0.6
Systemic hormonal preparations, excluding sex hormones	1.50 (1.8)	1.52 (1.9)	1.59 (1.9)	2.9
Anti-infectives for systemic use	6.60 (8.0)	6.27 (7.6)	6.50 (7.9)	−0.7
Antineoplastic and immunomodulating agents	0.82 (1.0)	0.86 (1.0)	0.90 (1.1)	4.7
Musculoskeletal system	4.24 (5.1)	4.12 (5.0)	4.09 (5.0)	−1.8
Nervous system	13.91 (16.8)	14.29 (17.4)	14.17 (17.2)	0.9
Antiparasitic	0.21 (0.3)	0.23 (0.3)	0.23 (0.3)	3.8
Respiratory system	5.85 (7.1)	5.94 (7.2)	6.01 (7.3)	1.4
Sensory organs	1.85 (2.2)	1.82 (2.2)	1.83 (2.2)	−0.7

* Mean percentage difference of medicaments consumption in period 2013–2015

In our opinion, a proper analysis of medication consumption is a basic prerequisite for regulatory decision-making and action.
